# Correction: Adherence to intravenous chemotherapy and associated factors among patients with cancer at Hawassa University Comprehensive Specialized Hospital Cancer Treatment Center, Sidama Region, Southern Ethiopia

**DOI:** 10.1371/journal.pone.0344481

**Published:** 2026-03-05

**Authors:** The PLOS One Staff

Table 5 was formatted incorrectly. Please see the correct Table 5 here.

The publisher apologizes for the error.

**Table 5:** Treatment-related characteristics of cancer patients under chemotherapy treatment at HUCSH, Hawassa, Sidama Regional State of Ethiopia, 2024 (n = 413).

Variable	Adherence		COR (95% C. I)	P-Value	AOR (95% C.I)	P-Value
Poor	Good
**Marital status**						
Married	177	145	2.9(1.2,7)	0.015	3.2(1.3,8)	0.01
Single	24	21	3.1(1.5-8.7)	0.03	4.3(1.4,13.5)	0.07
Divorced	11	3	0.97(0.2,4.4)	0.9	1.2(0.2,5.8)	0.9
Widowed	25	7	1		1	
**Occupation**						
Unemployed	81	54	1		1	
Marchant	38	31	1.2(0.7,2)	0.5	1.7(0.8,3)	0.14
Govt employee	29	53	2.7(1.5.4.8)	0.001	2.4(1.3,4.5)	0.004
Private employe	54	19	0.5(0.3,1)	0.045	0.6(0.3,1.2)	0.14
Farmer	35	19	0.8(0.4,1.5)	0.5	1.1(0.5,2)	0.96
I**nsurance**						
Yes	142	63	0.4(0.2,0.5)	0.00	0.5(0.3,1.1)	0.06
No	97	113	1		1	
**Transportation availability**						
Available	155	78	2.4(1.6, 3.5)	0.00	5.5(2.1,14)	0.01
Not available	82	98	1		1	
**Pain**						
Yes	38	13	1		1	
No	199	163	0.4(0.1,0.3)	0.00	0.4(0.2,1)	0.01
**Goal of treatment**						
Curative	68	73	1.8(1.2,2.6)	0.007	1.7(1.1,2.7)	0.02
Palliative	168	103	1		1	
**Social support**						
Good	154	83	2.3(1.7,8.5)	0.04	2.1(1.9,11.3)	0.04
Poor	79	100	1		1	
