# Supplementary material for: Adherence to intravenous chemotherapy and associated factors among patients with cancer at Hawassa University Comprehensive Specialized Hospital Cancer Treatment Center, Sidama Region, Southern Ethiopia
Source: PLoS One. 2025 Apr 7;20(4):e0321306. doi: 10.1371/journal.pone.0321306 (PMC11975084; doi:10.1371/journal.pone.0321306)
Supplement: Attachment — Submitted filename: Point-by-point resonse reviewers comment.docx [file pone.0321306.s004.docx]

January, 5, 2025

Tebelay Dilnessa, MSc
Academic Editor
PLOS ONE

Re: Submission of revised manuscript PONE-D-23-23059

Dear Ms. Tebelay Dilnessa and reviewers,

We greatly appreciate the reviewers for their constructive comments and suggestions. Please find the attached point-by-point response letter to the reviewers’ comments on the manuscript “Adherence to chemotherapy and associated factors among patients with cancer at Hawassa University Comprehensive Specialized Hospital Cancer Treatment Center, Sidama Region, Southern Ethiopia” with PONE-D-24-52523, for consideration. We have carefully reviewed the comments and we have corrected the manuscript accordingly. We hope that the revised version is now suitable for publication, and we look forward to hearing from you in due course.

Sincerely,

Bargude Balta (MSc, MPH)

Corresponding Author

Hawassa University college of health science Cancer Center

Hawassa, Ethiopia

Email: [barjuda@gmail.com](mailto:barjuda@gmail.com)

WhatsApp: +251919739646

**Point-by-Point Responses to Editors' Comments**

1. The ‘introduction’ should be replaced by ‘Background’.

Response to 1: Thank you editor for requesting revision. We replaced the introduction with a background

2. ………………… employed from May 20, 2024, to August 31, 2024.

**Response to 2**: We apologize for our mistake; we paraphrased

3. It is better to use formal and uniform use of AOR, 95%CI throughout the document including the abstract.

Response 3: Thank you, editor, for your comment we edited AOR throughout the document

4. In the abstract and result, the absolute number (numerator and denominator) is needed together with the percentage—for example, A/B (C%).

Response 4: Thank you very much editor we revised based on your comments

5. Your conclusion: - It's important to educate on the impact of non-adherence on survival and disease progression in palliative chemotherapy patients. Have you assessed the knowledge of study participants

Response 5: Thank you, editor, for your constructive comments, we revised the conclusion section of the abstract.

6. Your ‘Data availability’ statement: - All relevant data are within the manuscript and its supporting information files. If so, please attach a supporting file as a ‘supplementary file’.

Response 6: Thank you for asking for revision, this section was revised in both the document and submission system

**Introduction**

7. In the introduction part, the rationale is not explained well. Why you did do this research? What has been done before, what is known before, and which do you intend to fill?

Response 7: Thank you very much for your requests; we revised the rationale of the study

**Point-by-point responses to reviewers’ 1 questions and comments**

1. Abstract methods:

You mentioned that your sampling method was purposive. Would a non-randomized sampling technique enable you to make inferences?

Response 1: While a non-randomized sampling technique may limit the generalizability of findings to the broader cancer patient population, it remains a valuable approach for studying adherence to chemotherapy and its associated factors. In this study, a non-randomized sampling method was chosen to ensure the inclusion of patients actively undergoing chemotherapy, allowing for a more focused examination of adherence patterns and influencing factors. Given the nature of adherence studies, randomization may not always be feasible, as patient selection often depends on treatment accessibility, willingness to participate, and healthcare facility settings. We applied rigorous inclusion and exclusion criteria to minimize potential biases, ensured sample diversity, and used statistical adjustments to control for confounding variables. While our findings provide meaningful insights into adherence behavior, we acknowledge the need for cautious interpretation and recommend further research with randomized approaches to enhance external validity.

2. You have employed statistical analysis via multivariate analysis. However, it should be written as a multivariable analysis.

Response 2: thank you. We corrected it.

3. Abstract conclusion: The conclusion needs to be written based on your objectives. Therefore, you should first address the conclusion regarding the magnitude of chemotherapy adherence, followed by the findings related to your second objective of identifying factors associated with adherence. Finally, based on your findings, discuss the clinical and public health implications. Accordingly, your conclusion requires further improvement. Additionally, please rewrite the conclusion to ensure it aligns with your findings and compares them with international, regional, and national plans and actual experiences.

Response 3: Thank you, reviewer, we revised the entire conclusion

4. Your introduction section was written poorly and needs much improvement.
   Each paragraph is too long. Please reduce each to 3–4 lines, making the content short and precise. You did not follow the appropriate coherence for the statement of the problem. The first paragraph should define the problem, the second should address the severity of the problem from a global to a national perspective, the third should discuss the actual and potential burden, the fourth should outline the factors associated with adherence, and the fifth should detail intervention experiences concerning the problem. Finally, the research gap should be identified to convince the scientific community, and the rationale for your study should be well-articulated.

Response 4: Thank you very much reviewer for your constructive comments: we revised the entire introduction

5. A study design: that voids redundancy words and can be written as an institutional-based cross-sectional study was employed.

Response 5: we appreciate this feedback and have revised

6. Source of population; Please do not limit the source population to only the Sidama region. Consider including patients from the Guji and Western Arsi zones in the Oromia region and other referral areas in southern Ethiopia. Therefore, ensure it reflects the actual experience.

Response 6: Thank you, reviewer, I appreciate your effort, I fully accepted and revised the source population

7. Sample size and sampling technique: if you have included all patients in the cancer center no need to estimate the sample size. I need an explanation does Purposive sampling enable us to make inferences?

Response 7: Purposive sampling, while not a probability-based method, can still enable meaningful inferences, particularly in studies focusing on specific populations, such as cancer patients undergoing chemotherapy. In our study, purposive sampling was employed to ensure the inclusion of eligible participants actively receiving treatment at the cancer center, allowing us to examine adherence patterns and associated factors within this defined group. This approach enhances the study's internal validity by capturing relevant data from those directly experiencing the phenomenon under investigation. However, we acknowledge that purposive sampling may limit external validity and generalizability to broader cancer populations. To mitigate this, we ensured sample diversity and applied statistical techniques to address potential biases. While probability sampling would strengthen generalizability, purposive sampling remains an appropriate choice for in-depth analysis of adherence behaviors in a targeted clinical setting."

8. Social support needs more explanation, including the scoring system and the criteria for classifying patients as having good or poor social support.

Response 8: Thank you, reviewer, for your request for social support. we used the MSPSS tool, which has 12 questions. We used to mean of 12 questions those who responded above the mean indicated greater perceived social support and those below the mean coded as poor social support. And indicated under the operational definition.

Result
9. Please make the sociodemographic text results concise.

Response 9: Thank you very much for the comments we edited sociodemographic texts

10. What are your bases for classifying monthly income?

Response 9; Thank you, reviewer, for your request on the monthly income category, we categorized it based similar study baseline. We used the category of World Bank income categorization of 2024. Hence (category of WB annual income: low income, <=$995; lower middle income, $996–3,945; upper middle income, $3,946–12,195; and high income, $12,196 or more.). Based on our data the category is low-income and lower-middle-income, hence in our case there is no upper-middle-income and high-income category.

11. Your tables did not comply with the journal's standards. Please, comply with journal standards.

Response 11: Thank you editor your genuine comments, we edited tables respect with to journal guideline
12. The social support table needs improvement, such as writing the research question in a shorter, more precise manner, and ensuring it is suitable for the readers.

Response 12: Thank you, reviewers, The questions under the tool MSPSS we used for social support were somewhat longer; by considering the originality of the tool we minimized some words suitable for readers.
13. Magnitude of adherence: please write and report only the good adherence.
Response 13; Thank you very much reviewer for your comment we revised the magnitude of the adherence report

Discussion

14.. The introductory statement of the discussion needs to be impressive. First, you need to discuss your first objective finding. write the possible explanation of why your figure is higher or lower or similar with other results.

Your discussion of the second objective (factors) was very poor. First, compare your findings with those of others, then provide possible explanations, and finally, discuss the clinical and public health implications.

Response 14: Response: we appreciate this feedback and have reviewed the entire discussion and edited it.

14. Make it compliant with the journal standard and include DOI number.

Response 14: Thank you editor for your request to edit the journal standard and DOI. We involved DOI for all references except books and reports.

**Point-by-point responses to Reviewer #2** **questions and comments**

1. The definition of adherence needs to be spelled out, is it adherence to systemic IV chemotherapy? adherence to oral chemotherapy? both? and why if systemic chemotherapy was not taken from the chart (as no-shows) but with the Morisky tool as clearly to recruit these patients they had to be in the hospital?

Response 1: Thank you, reviewer, for your detailed comments. We revised the definition of adherence to **Adherence to IV chemotherapy:**  and took patient data from those admitted to inpatient care for intravenous chemotherapy. That means from both the patients and patient chart. Under the data collection procedure section, we included data collected from patient charts and interviews.

2. Throughout the paper it is not defined and that is crucial for understanding adherence to what?

Response 2: Thank you very much we operationalized adherence to IV chemotherapy.

3. In terms of study data collection, there are a lot of variables described, but only for social support the tool is described how it is measured. Please include how each of these variables were measured and used in the analyses.

Response 3: "We appreciate the reviewer's insightful comment. We edited the entire data collection section. A detailed description of measurement tools and variable categorization has been included in the revised manuscript for clarity.

4. Also please clarify the consent process, it is not in the paper, but the ethics statement is confusing, saying written consent was obtained as well as saying consent was not required as it was a retrospective chart review. And that also needs to be clarified in the methods, as clearly data came from the chart.

Response 4: Thank you, editor, for your comments we apologize for our mistakes, we interviewed patients so we took consent from the patient and removed this part from ethics “Consent was not required as it was a retrospective chart review”

5. I would strongly suggest you use a native speaker to review the paper. There are many unclear sentences and the tense should be past tense (the study is completed) and it is written like a protocol, that the work still needs to happen. There are also a lot of duplicate sentences, such as the patient population, study subjects, and inclusion criteria, it is all the same information repeated three times. So please review

Response 5: Thank you, reviewer, we revised the entire manuscript for language and any related editing

6. In terms of the background, you refer to other studies but no references are provided. So please review and add why not. And I think it could be more succinct.

Response 6: Thank you very much, we revised the entire background.

7. On page 2 there is a whole section on pts stopped appropriately, that paragraph needs to be reviewed as it is unclear what you mean for me. In terms of the study area, I think some of the details can go, it is irrelevant what latitude, etc the hospital is at.

Response 7: Thanks, reviewer, for your comments, we reviewed the methodology and removed unnecessary descriptions.

8. The sample size calculation section could be more clearly worded, it seems it is based on another study but it is not completely clear to me for sampling it says all patients were included in the period, but the period is not described, and did no one decide to refuse to participate?

Response 8: Thank you very much reviewer we used the proportion of chemotherapy adherence done in a previous study in the Amhara region Ethiopia, to compute the sample size calculation and we cited it. Regarding the study period, we mentioned it as a study area and the period section. Our study period was “May 20 to August 31, 2024”. All of our respondents responded survey

9. And for the analysis, it is not multivariate but multiple variable logistic regression analysis. Can you please describe how you decided which variables in what order you put them in the multiple variable model? What were the model fit statistics as there is no AUC/ ROC or anything reported?

Response 9: **Selection of Variables for the Multiple Variable Logistic Regression Model:**
Variables were selected for inclusion in the multiple variable logistic regression model based on their clinical relevance, statistical significance in the bivariate analysis (p-value ≤ 0.25), and prior evidence from existing literature. Initially, a bivariate logistic regression was conducted to identify candidate variables associated with the case magnitude. Variables with a significant association at the bivariate level, along with key demographic and risk factors known to influence adherence, were included in the multiple variable models.

**Order of Variable Inclusion:**

A hierarchical approach was used, beginning with demographic factors (such as marital status, and occupation), followed by other factors such as insurance and Transportation availability This stepwise approach ensured that potential confounders were controlled while assessing the independent effects of each variable.

**Model Fit Statistics:**

Although AUC/ROC analysis was not explicitly reported, model fit was assessed using statistical measures such as the Hosmer-Lemeshow goodness-of-fit test to evaluate calibration, and the likelihood ratio test to compare nested models. Additionally, the Nagelkerke R² was used to estimate the proportion of variance explained by the model. If required, further model discrimination metrics such as AUC/ROC can be computed to strengthen the validity of the findings.

10. And for table 1 why are all aged 51 and over grouped?
    For the monthly income can you report it in US dollars for all readers?

Response 10; Thank you reviewer we have very few patients above 60 so we merged them and categorized ages above 50. We changed the monthly income to US dollars for all reviewers.

11. How was missing data handled?

Response 11: Missing data was handled using appropriate statistical techniques to minimize potential biases. Cases with incomplete or missing key variables were carefully examined, and where feasible, missing values were addressed through multiple imputation or mean substitution methods. Since our missing data were minimal a complete case analysis was performed to ensure the integrity of the findings. Additionally, sensitivity analyses were conducted to assess the impact of missing data on the study results. Efforts were also made during data collection to minimize missing responses by providing thorough training to data collectors and conducting real-time data checks."

12. For the social support table, I think you can keep the percentage and remove the frequency but I am not sure why this data is presented as the variable was categorized in the statistical analyses.

Response 12: Thank you, reviewer, we used the MSPSS tool to measure social support, it has 12 questions/items and reported results. Then we did mean and categorized it as good and poor social support based on the mean. above the mean good social support and below the mean poor social support. Also, we removed frequency.

13. For Table 6 there are some cells with <10 persons, those categories need to be collapsed with others, why is income not included in these models? in the introduction, you describe how income is the most important factor for adherence in similar studies in Africa and how it was collected.

Response 13: Thank you, reviewer, we acknowledge the comments, for each cell more than 5 observations were collapsed in the revised version. Regarding monthly income, we checked it in the bivariable regression cut point p-value of 0.25 but it is not a candidate variable for multivariable regression so we didn’t include it in the factor table even though it is an important factor in Africa.

14. I think the discussion and conclusion need to be looked at after the English has been corrected, there are too many sentences unclear to me.
15. Response 14 Thank you reviewer I appreciate your effort for scientific advances we edited the entire manuscript.
