# Supplementary material for: Adherence to intravenous chemotherapy and associated factors among patients with cancer at Hawassa University Comprehensive Specialized Hospital Cancer Treatment Center, Sidama Region, Southern Ethiopia
Source: PLoS One. 2025 Apr 7;20(4):e0321306. doi: 10.1371/journal.pone.0321306 (PMC11975084; doi:10.1371/journal.pone.0321306)
Supplement: Attachment — Submitted filename: plose one.docx [file pone.0321306.s001.docx]

Thank you for giving me the opportunity to participate as a reviewer.

**Generally**

your manuscript is good. Has many grammar, clarity, and fluency issues. However, the authors need to improve their work and make it suitable for the scientific community in a short period by positively addressing the reviewers' comments.

**Title**

The title is very important as it addresses the community, the severity of cancer, and government-related issues.

**Abstract**

Generally, well structured

Abstract methods:

- You mentioned that your sampling method was purposive. Do you think a non-randomized sampling technique enables you to make inferences?
- You have employed the statistical analysis via multivariate analysis. However, it should be written as multivariable analysis.

Abstract conclusion:

The conclusion needs to be written based on your objectives. Therefore, you should first address the conclusion regarding the magnitude of chemotherapy adherence, followed by the conclusions related to your second objective of identifying factors associated with adherence. Finally, based on your findings, discuss the clinical and public health implications. Accordingly, your conclusion requires further improvement. Additionally, please rewrite the conclusion to ensure it aligns with your findings and compares them with international, regional, and national plans and actual experiences.

**Introductions**

Your introduction section was written in poor way and need much improvement.

Each paragraph is too long. Please reduce each to 3–4 lines, making the content short and precise.

You did not follow the appropriate coherence for the statement of the problem. The first paragraph should define the problem, the second should address the severity of the problem from a global to a national perspective, the third should discuss the actual and potential burden, the fourth should outline the factors associated with adherence, and the fifth should detail intervention experiences concerning the problem. Finally, the research gap should be clearly identified to convince the scientific community, and the rationale for your study should be well-articulated.

**Methods and materials**

A study design: voids redundancy words and can be written as institutional based cross-sectional study was employed.

Source of population; Please do not limit the source population to only the Sidama region. Consider including patients from the Guji and Western Arsi zones in the Oromia region and other referral areas in southern Ethiopia. Therefore, ensure it reflects the actual experience.

Sample size and sampling technique: if you have included all patients in cancer center no need of estimating sample size. I need explanation that does Purposive sampling enables us to make inference?

Social support needs more explanation, including the scoring system and the criteria for classifying patients as having good or poor social support.

**Result**

- Please make the sociodemographic text results concise.
- What are your bases for classifying monthly income?
- Your tables did not comply with the journal's standards. Please, comply with journal standard.
- The social support table needs improvement, such as writing the research question in a shorter, more precise manner, and ensuring it is suitable for the readers.
- Magnitude of adherence: please write and report only the good adherence.
- You did not write statistical interpretation in the factor report.

**Discussion**

- The introductory statement of the discussion needs to impressive.
- First, you need to discuss your first objective finding.
- write the possible explanation of why your figure is higher or lower or similar with other results
- Your discussion of the second objective (factors) was very poor. First, compare your findings with those of others, then provide possible explanations, and finally, discuss the clinical and public health implications.

**Conclusion**

- The conclusion statement needs to be written by comparing your results with the plans and outcomes of international, regional, and national experiences. Therefore, avoid the published result.
- You have included too many unnecessary personal opinions. Therefore, the recommendation statement should be based on your findings.

**References**

Make it compliance with the journal standard and includes DOI number.
