# Supplementary material for: Adherence to intravenous chemotherapy and associated factors among patients with cancer at Hawassa University Comprehensive Specialized Hospital Cancer Treatment Center, Sidama Region, Southern Ethiopia
Source: PLoS One. 2025 Apr 7;20(4):e0321306. doi: 10.1371/journal.pone.0321306 (PMC11975084; doi:10.1371/journal.pone.0321306)
Supplement: Attachment — Submitted filename: plose chemotheraphy adhrence.docx [file pone.0321306.s003.docx]

**Second round comments**

The manuscript has improved following the revision.

**Title**

Need to include IV

**Introduction**

The introduction requires improvement, including the addition of the following elements: the magnitude of chemotherapy adherence, consolidation of burden statements into a single paragraph, discussion of factors associated with chemotherapy adherence, addressing interventions, and a clearer articulation of the research gap.

**Discussion**

The discussion need improvement particularly, clinical and public health implications

The possible explanation or speculation need citation
