# Supplementary material for: Adherence to intravenous chemotherapy and associated factors among patients with cancer at Hawassa University Comprehensive Specialized Hospital Cancer Treatment Center, Sidama Region, Southern Ethiopia
Source: PLoS One. 2025 Apr 7;20(4):e0321306. doi: 10.1371/journal.pone.0321306 (PMC11975084; doi:10.1371/journal.pone.0321306)
Supplement: Attachment — Submitted filename: Resonse to editor and reviewers comment.docx [file pone.0321306.s005.docx]

March, 1, 2025

Tebelay Dilnessa, MSc
Academic Editor
PLOS ONE

Re: Submission of revised manuscript PONE-D-23-23059

Dear Ms. Tebelay Dilnessa and reviewers,

We greatly appreciate the reviewers for their constructive comments and suggestions. Please find the attached point-by-point response letter to the reviewers’ comments on the manuscript “Adherence to chemotherapy and associated factors among patients with cancer at Hawassa University Comprehensive Specialized Hospital Cancer Treatment Center, Sidama Region, Southern Ethiopia” with PONE-D-24-52523, for consideration. We have carefully reviewed the comments and we have corrected the manuscript accordingly. We hope that the revised version is now suitable for publication, and we look forward to hearing from you in due course.

Sincerely,

Bargude Balta (MSc, MPH)

Corresponding Author

Hawassa University college of health science Cancer Center

Hawassa, Ethiopia

Email: [barjuda@gmail.com](mailto:barjuda@gmail.com)

WhatsApp: +251919739646

**Point-by-Point Responses to Editors' Comments**

1. The figure (s) should be prepared without description in the TIF version and uploaded as a separate file. The description should be put both within the main manuscript where the figure is cited and below the list of references.

Response to 1: Thank you editor for requesting revision. We uploaded it without description and placed it in the main manuscript where the figure is cited and after reference

2 The document should be proofread and keep its scientific quality.

**Response to 2**: We proofread the document while ensuring that its scientific quality and integrity are maintained."

3 The author's contribution should be removed from the main manuscript because the system already created it.

Response 3: Noted. The authors' contribution section will be removed from the main manuscript, as the system will generate it automatically."

**Point-by-point responses to Reviewer #1** **questions and comments**

**His comments:**

1. The introduction requires improvement, including the addition of the following elements: the

magnitude of chemotherapy adherence, consolidation of burden statements into a single

paragraph, discussion of factors associated with chemotherapy adherence, addressing

interventions, and a clearer articulation of the research gap.

Response 1: Thank you, reviewer, for revisiting and commenting on our manuscript we revised and addressed the issue in the 2^nd^ and 3^rd^ paragraphs.

2. The discussion needs improvement, particularly regarding clinical and public health implications. Possible explanations or speculations need to be cited.

Response 2: Thank you for your feedback. I agree that the discussion section could benefit from further elaboration on the clinical and public health implications. It is essential to contextualize the findings by exploring the potential impact on healthcare delivery and patient outcomes. We added two paragraphs under the discussion section before the limitation of study which can address the issue.

**Reviewer #2: Thank you for the revision. I would have preferred to see a tracked changes version. A lot of comments have not been appropriately addressed.**

Response: Thank you for your feedback. I acknowledge that some comments may not have been fully addressed, and I sincerely appreciate your patience. I will carefully review all the provided comments to ensure they are appropriately incorporated. If there are specific areas that require further clarification or revision, please let me know, and I will make the necessary improvements to strengthen the work. Your insights are valuable in enhancing the quality and rigor of the discussion. Here we revisited our revision reviewer comments there were highlights of revision in the main manuscript.

1. The definition of adherence needs to be spelled out, is it adherence to systemic IV chemotherapy? adherence to oral chemotherapy? both? and why if systemic chemotherapy was not taken from the chart (as no-shows) but with the Morisky tool as clearly to recruit these patients they had to be in the hospital?

Response 1: Thank you, reviewer, for your detailed comments. We acknowledge reviewer comments that The **Morisky Medication Adherence Scale (MMAS)** was originally designed to assess adherence to oral medications, particularly for chronic diseases like hypertension and diabetes. While it has been widely used in various medical contexts, its applicability to **intravenous (IV) chemotherapy adherence. We put previous studies done using the Morisky Medication Adherence Scale to measure adherence to IV chemotherapy. “**Bekalu YE, Wudu MA, Gashu AW. Adherence to Chemotherapy and Associated Factors Among Patients With Cancer in Amhara Region, Northeastern Ethiopia, 2022. A Cross-Sectional Study. Cancer Control. 2023;30:1–10.”

2. Throughout the paper it is not defined and that is crucial for understanding adherence to what?

Response 2: Thank you very much we operationalized adherence to IV chemotherapy (It has been highlighted in a manuscript operational definition section.

3. In terms of study data collection, there are a lot of variables described, but only for social support the tool is described how it is measured. Please include how each of these variables were measured and used in the analyses.

Response 3: "We appreciate the reviewer's insightful comment. We edited the entire data collection section. A detailed description of measurement tools and variable categorization has been included in the revised manuscript for clarity. **We highlighted under the data collection tool section**

4. Also please clarify the consent process, it is not in the paper, but the ethics statement is confusing, saying written consent was obtained as well as saying consent was not required as it was a retrospective chart review. And that also needs to be clarified in the methods, as clearly data came from the chart.

Response 4: Thank you, reviewer, for your comments we apologize for our mistakes, we interviewed patients so we took consent from the patient and removed this part from ethics “Consent was not required as it was a retrospective chart review” and revised the remaining section

5. I would strongly suggest you use a native speaker to review the paper. There are many unclear sentences and the tense should be past tense (the study is completed) and it is written like a protocol, that the work still needs to happen. There are also a lot of duplicate sentences, such as the patient population, study subjects, and inclusion criteria, it is all the same information repeated three times. So please review

Response 5: Thank you, reviewer, we revised the entire manuscript for language and any related editing

6. In terms of the background, you refer to other studies but no references are provided. So please review and add why not. And I think it could be more succinct.

Response 6: Thank you very much, we revised the entire background.

7. On page 2 there is a whole section on pts stopped appropriately, that paragraph needs to be reviewed as it is unclear what you mean for me. In terms of the study area, I think some of the details can go, it is irrelevant what latitude, etc the hospital is at.

Response 7: Thanks, reviewer, for your comments, we reviewed the methodology and removed unnecessary descriptions.

8. The sample size calculation section could be more clearly worded, it seems it is based on another study but it is not completely clear to me for sampling it says all patients were included in the period, but the period is not described, and did no one decide to refuse to participate?

Response 8: Thank you very much reviewer we used the proportion of chemotherapy adherence done in a previous study in the Amhara region Ethiopia, to compute the sample size calculation and we cited it. Regarding the study period, we mentioned it as a study area and the period section. Our study period was “May 20 to August 31, 2024”. All of our respondents responded survey

9. And for the analysis, it is not multivariate but multiple variable logistic regression analysis. Can you please describe how you decided which variables in what order you put them in the multiple variable model? What were the model fit statistics as there is no AUC/ ROC or anything reported?

Response 9: **Selection of Variables for the Multiple Variable Logistic Regression Model:**
Variables were selected for inclusion in the multiple variable logistic regression model based on their clinical relevance, statistical significance in the bivariate analysis (p-value ≤ 0.25), and prior evidence from existing literature. Initially, a bivariate logistic regression was conducted to identify candidate variables associated with the case magnitude. Variables with a significant association at the bivariate level, along with key demographic and risk factors known to influence adherence, were included in the multiple variable models.

**Order of Variable Inclusion:**

A hierarchical approach was used, beginning with demographic factors (such as marital status, and occupation), followed by other factors such as insurance and Transportation availability This stepwise approach ensured that potential confounders were controlled while assessing the independent effects of each variable.

**Model Fit Statistics:**

Although AUC/ROC analysis was not explicitly reported, model fit was assessed using statistical measures such as the Hosmer-Lemeshow goodness-of-fit test to evaluate calibration, and the likelihood ratio test to compare nested models. Additionally, the Nagelkerke R² was used to estimate the proportion of variance explained by the model. If required, further model discrimination metrics such as AUC/ROC can be computed to strengthen the validity of the findings.

10. And for table 1 why are all aged 51 and over grouped?
    For the monthly income can you report it in US dollars for all readers?

Response 10; Thank you reviewer we have very few patients above 60 so we merged them and categorized ages above 50. We changed the monthly income to US dollars for all reviewers.

11. How was missing data handled?

Response 11: Missing data was handled using appropriate statistical techniques to minimize potential biases. Cases with incomplete or missing key variables were carefully examined, and where feasible, missing values were addressed through multiple imputation or mean substitution methods. Since our missing data were minimal a complete case analysis was performed to ensure the integrity of the findings. Additionally, sensitivity analyses were conducted to assess the impact of missing data on the study results. Efforts were also made during data collection to minimize missing responses by providing thorough training to data collectors and conducting real-time data checks."

12. For the social support table, I think you can keep the percentage and remove the frequency but I am not sure why this data is presented as the variable was categorized in the statistical analyses.

Response 12: Thank you, reviewer, we used the MSPSS tool to measure social support, it has 12 questions/items and reported results. Then we did mean and categorized it as good and poor social support based on the mean. above the mean good social support and below the mean poor social support. Also, we removed frequency.

13. For Table 6 there are some cells with <10 persons, those categories need to be collapsed with others, why is income not included in these models? in the introduction, you describe how income is the most important factor for adherence in similar studies in Africa and how it was collected.

Response 13: Thank you, reviewer, we acknowledge the comments, for each cell more than 5 observations were collapsed in the revised version. Regarding monthly income, we checked it in the bivariable regression cut point p-value of 0.25 but it is not a candidate variable for multivariable regression so we didn’t include it in the factor table even though it is an important factor in Africa.

14. I think the discussion and conclusion need to be looked at after the English has been corrected, there are too many sentences unclear to me.

Response 14: Thank you, reviewer, I appreciate your effort for scientific advances we edited the entire manuscript.
